# Influence of maternal and perinatal factors on subsequent hospitalisation for asthma in children: evidence from the Oxford record linkage study

**DOI:** 10.1186/1471-2466-10-14

**Published:** 2010-03-16

**Authors:** Rebekah Davidson, Stephen E Roberts, Clare J Wotton, Michael J Goldacre

**Affiliations:** 1 Unit of Health-Care Epidemiology, Department of Public Health, University of Oxford, Old Road Campus, Old Road, Oxford OX3 7LF, UK; 2 School of Medicine, Swansea University, Singleton Park, Swansea, SA2 8PP, UK

## Abstract

**Background:**

There is much interest in the possibility that perinatal factors may influence the risk of disease in later life. We investigated the influence of maternal and perinatal factors on subsequent hospital admission for asthma in children.

**Methods:**

Analysis of data from the Oxford record linkage study (ORLS) to generate a retrospective cohort of 248 612 records of births between 1970 and 1989, with follow-up to records of subsequent hospital admission for 4 017 children with asthma up to 1999.

**Results:**

Univariate analysis showed significant associations between an increased risk of admission for asthma and later years of birth (reflecting the increase in asthma in the 1970s and 1980s), low social class, asthma in the mother, unmarried mothers, maternal smoking in pregnancy, subsequent births compared with first-born, male sex, low birth weight, short gestational age, caesarean delivery, forceps delivery and not being breastfed. Multivariate analysis, identifying each risk factor that had a significant effect independently of other risk factors, confirmed associations with maternal asthma (odds ratio (OR) 3.1, 95% confidence interval 2.7-3.6), male sex (versus female, 1.8, 1.7-2.0), low birth weight (1000-2999 g versus 3000-3999 g, 1.2, 1.1-1.3), maternal smoking (1.1, 1.0-1.3) and delivery by caesarean section (1.2; 1.0-1.3). In those first admitted with asthma under two years old, there were associations with having siblings (e.g. second child compared with first-born, OR 1.3, 1.0-1.7) and short gestational age (24-37 weeks versus 38-41 weeks, 1.6, 1.2-2.2). Multivariate analysis confined to those admitted with asthma aged six years or more, showed associations with maternal asthma (OR 3.8, 3.1-4.7), age of mother (under 25 versus 25-34 at birth, OR 1.16, 1.03-1.31; over 35 versus 25-34, OR 1.4, 1.1-1.7); high social class was protective (1 and 2, compared with 3, 0.72; 0.63-0.82). Hospital admission for asthma in people aged over six was more common in males than females (1.4; 1.2-1.5); but, by the teenage years, the sex ratio reversed and admission was more common in females than males.

**Conclusion:**

Several maternal characteristics and perinatal factors are associated with an elevated risk of hospital admission for asthma in the child in later life.

## Background

Asthma is characterised by widespread reversible narrowing of the bronchioles leading to dyspnoea, cough and wheezing. In some cases it may be brought on by exposure to a particular, identified allergen but in many cases there is no single identified causal factor. Much is still unknown about its aetiology or about factors associated with susceptibility to it. With high prevalence of asthma across the developed world [1], there is a continued need for research into potential risk factors.

Previously documented risk factors for asthma include maternal asthma [2,3], maternal smoking, [2,4] low birth weight and short gestational age [2,5-7]. It has also been suggested that caesarean delivery may increase the risk [8], and that breastfeeding may reduce the risk, of asthma in the child [9]. Studies into the risk of asthma associated with some other perinatal factors and maternal characteristics have been inconclusive.

The aims of this study were to investigate associations between maternal and perinatal risk factors and the subsequent hospitalisation for asthma in the child. We used record linkage data from a large geographically defined English population. We report findings for 4 017 children with a history of hospital admission or day case care for asthma, compared with 244 595 children with no such history.

## Methods

We used the dataset of the Oxford record linkage study (ORLS). The ORLS includes hospital inpatient records (including admissions for inpatient day case care) in an area of South East England. Standard data collection - data very similar to the Hospital Episode Statistics collected elsewhere in England - was undertaken in two health districts of Oxfordshire and West Berkshire from 1963 to 1999 (population 0.9 million in 1999) and, from 1975 to 1999, it covered a further four adjacent districts (total population 1.9 million). A specialist data collection system for maternity - used as the basis of this study - covered all births in National Health Service (NHS) hospitals in Oxfordshire and West Berkshire from 1970 to 1989.

The maternity data from 1970-1989 were abstracted from maternity records by clerical staff who were trained by senior medical staff at the ORLS. The records of each mother and her baby were routinely linked by the use of unique numbers for each mother and each baby that were assigned to both the baby's and the mother's record. Detailed data collection on maternities in the ORLS area stopped in 1989 following government reforms to increase the uniformity of NHS data collection systems. Data collection for general hospital admissions in the ORLS area continued but, with further changes to NHS information systems in 1999, it is not possible to link pre- and post-1999 ORLS data.

We identified cases of asthma in children and their mothers, from hospital admission records, using ICD (International Classification of Diseases) codes 493 in the 8^th ^and 9^th ^revisions, and J45 and J46 in ICD-10. We analysed the data for children of all ages together; and we also subdivided the analysis by age into those who were first admitted with asthma under the age of 2 years, first admitted aged 2-10 years and aged over 11 years. We studied those first admitted under 2 years separately because a diagnosis of asthma made in this age group is sometimes less certain than that made in older children; and because possible influences of perinatal factors on true asthma may be more evident in those diagnosed young. We studied those aged 11 and over because, given that there are only ten years of 'follow up' for the youngest children, we wanted to be sure that differences in duration of 'follow up' had little or no effect in our findings. They did not, and so for brevity we do not present findings separately on those aged 11 and over.

The variable 'social class' is occupation-based social class. It was coded, at the original time of data collection and coding, based on the occupation of the head of household (for a child), the occupation of the patient if an adult male, the occupation of the husband of a married woman, or the woman's own occupation if a single woman. This was the standard practice used at the time in the English national decennial census run by the Office of Population Censuses and Surveys (OPCS). The ORLS staff collected the data in the same way and coded occupational data and its associated social class according to the occupational coding manual of the OPCS. The standard social classes have the summary terminology of: I Professional, II Managerial, III Skilled manual and non-manual workers, IV Partly skilled, V Unskilled. Thus, in socio-demographic status, they range from I at the top of the hierarchy to V at the bottom.

There were 253 021 pregnancies with a maternity record in the ORLS from 1970-1989. Of these, we excluded 985 that were recorded as having ended in abortion, 1 560 stillbirths and 1 566 neonatal deaths. We also excluded 289 maternities in which the birth weight was recorded as less than 1000 grams because many of these records had implausibly low values and/or considerable missing data for some of the other variables. After applying these exclusion criteria, there were 248 612 children in the study population.

Initial analyses were undertaken on fine groupings of the maternal and perinatal variables and, following scrutiny, broader groupings - as shown in the tables - were selected for further analysis. Some of the variables - maternal smoking, social class and breastfeeding - were not recorded in the first five years covered by this study.

The significance of univariate associations was tested using chi-square tests. When using logistic regression for multivariate modelling, all variables that were significant (p < 0.05) in the univariate analysis were included in the initial model and the variables that were not significant were removed. In further modelling, each variable that was not significant in univariate analysis was re-introduced, one at a time, into the model. The purpose of this was to test whether any variable, not significant in univariate analysis, became so when modelled with the other significant variables.

Approval for the research programme of the Unit of Health-Care Epidemiology using the anonymised dataset of the Oxford Record Linkage Study has been obtained from the Central and South Bristol Research Ethics Committee (04/Q2006/176).

## Results

There were 248 612 births in the study. Of these, 4 017 children had a subsequent hospital admission or hospital-based day case care for asthma. Of the 4 017, 535 (13.3%) had a record of first admission aged under two years of age, 2 793 (69.5%) at 2-10 and 689 (17.2%) at the age of 11 or over (Table 1). 62.6% of those with asthma were males (Table 1).

**Table 1:** Number of children admitted for asthma based on age at admission and sex.

Age at asthma admission	Male	Female	Total
**0-1**	374	161	535

**2-10**	1838	955	2793

**11+**	304	385	689

**Total**	2516	1501	4017

The cumulative incidence of hospitalised asthma, overall, was 1.62% of all children; and the cumulative incidence by the age of ten years was 1.34%. Asthma was more common in children born in later birth cohorts. The percentages of children with asthma aged 2 or less, in each successive five-year period from 1970-4 to 1985-9, were 0.03%, 0.07%, 0.21% and 0.55%. The respective percentages for asthma aged 2 and over, in the four consecutive periods, were 1.07%, 1.47%, 1.69% and 1.43%.

### Incidence of hospitalised asthma associated with each factor: Table 2

Maternal asthma was a risk factor for asthma in the child in both broad age groups: 9% of the children with asthma under 2, 8% of those aged 2 and over, and 2% with no record of asthma, had mothers with a record of hospital admission for asthma (Table 2). Low social class, unmarried motherhood, maternal smoking in pregnancy, short gestational age, low birth weight, caesarean delivery, and male sex were all significant predictors of asthma in the child, both in those with a first admission aged under 2 and in those first admitted at an older age (Table 2). A significant but modest effect was also found for maternal age (slightly elevated for children of mothers aged under 25 or aged 35 and over). Details of the risk factors in the individual age groups are shown in Table 2. Factors significantly associated with asthma, but only in those under 2, were being first-born (which was protective: 36% of those with an admission for asthma under 2 were first born, compared with 41% of those aged 2 or over and 42% of those without asthma); forceps delivery; and being breastfed at the time of leaving hospital. Maternal age and Apgar 1 were significantly associated with asthma in those aged 2 and over but not in those younger.

**Table 2:** Percentage distribution of values of each factor in each group of children, with chi-square tests of association

	% of each group of children				Chi^2 ^(df), with p value below		
	**All asthma** **(N = 4017)**	**Asthma, aged 0-1** **(N = 535)**	**Asthma, aged 2+** **(N = 3482)**	**No asthma** **(N = 244595)**	**All asthma**	**Asthma aged 0-1**	**Asthma aged 2+**

**Maternal asthma:**							

No	92.0	91.0	92.2	97.6	495.6(1)	96.2(1)	409.1(1)

Yes	8.0	9.0	7.8	2.4	0.0001	<0.0001	0.0001

Total	100	100	100	100			

**Year of birth:**							

1970-1974	18.9	4.1	21.1	27.9	200.4(3)	497.9 (3)	94.1(3)

1975-1979	21.7	7.7	23.8	22.7	<0.0001	<0.0001	0.0001

1980-1984	28.4	23.2	29.2	24.2			

1985-1989	31.0	65.0	25.8	25.2			

Total	100	100	100	100			

							

**Maternal age:**							

14-24	37.3	38.5	37.1	34.8	11.8(2)	4.0 (2)	10.2(2)

25-34	55.0	55.3	54.9	57.6	0.003	0.14	0.006

35-49	7.7	6.2	8.0	7.6			

Total	100	100	100	100			

							

**Social class:**							

I + II	30.2	29.7	30.2	35.9	48.6(2)	7.4(2)	41.5(2)

III	48.2	48.1	48.2	45.5	<0.0001	0.025	0.0001

IV + V	21.6	22.2	21.6	18.6			

Total	100	100	100	100			

							

**Marital status:**							

Married	88.0	79.4	89.4	90.4	25.3(1)	74.0(1)	4.3(1)

Not married	12.0	20.6	10.6	9.6	<0.0001	0.0001	0.04

Total	100	100	100	100			

							

**Parity of mother:**							

0	40.5	35.5	41.2	42.0	14.0(4)	24.2(4)	4.65(4)

1	35.4	35.3	35.3	35.9	0.007	<0.0001	0.33

2	15.3	16.8	15.1	14.5			

3	5.9	8.6	5.5	4.9			

4+	2.9	3.6	2.9	2.7			

Total	100	100	100	100			

							

**Maternal smoking:**							

No	71.7	63.3	73.4	76.5	34.9(1)	46.0(1)	11.7(1)

Yes	28.3	36.7	26.6	23.5	0.0001	0.0001	0.0006

Total	100	100	100	100			

							

**Gestational age (wks):**							

24-37	12.8	18.0	12.1	10.1	43.2(3)	33.2 (2)	13.3(2)

38-41	77.4	70.7	78.3	80.4	0.0001	0.0001	0.001

42-47	9.8	11.3	9.6	9.5			

Total	100	100	100	100			

							

**Birth weight:**							

1000-2999	27.1	32.0	26.3	23.6	27.4(2)	22.5 (2)	16.1(2)

3000-3999	64.4	61.8	64.8	67.9	0.0001	0.0001	0.0003

4000-5499	8.5	6.2	8.9	8.5			

Total	100	100	100	100			

							

**Caesarean delivery:**							

No	91.0	89.8	91.2	92.6	14.1(1)	5.7(1)	9.7(1)

Yes	9.0	10.2	8.8	7.4	0.0002	0.02	0.002

Total	100	100	100	100			

							

**Forceps delivery:**							

No	88.2	93.4	87.4	86.9	5.9(1)	18.5(1)	1.0(1)

Yes	11.8	6.6	12.6	13.1	0.01	0.0001	0.33

Total	100	100	100	100			

							

**Apgar 1:**							

1-5	8.4	9.3	8.2	9.5	9.4(2)	2.1(2)	8.5(2)

6-8	30.3	31.3	30.2	28.48	0.01	0.35	0.01

9-10	96.1	59.4	61.6	62.5			

Total	100	100	100	100			

							

**Number of babies in birth delivery:**							

Singleton	98.1	97.6	98.2	97.8	1.7(1)	0.2(1)	2.9(1)

Multiple birth	1.9	2.4	1.8	2.2	0.19	0.68	0.09

Total	100	100	100	100			

							

**Sex of baby:**							

Female	32.4	30.1	38.5	48.8	205.9(1)	74.6(1)	145.5

Male	67.4	69.9	61.5	51.2	<0.0001	0.0001	0.0001

Total	100	100	100	100			

							

**Breastfed or not:**							

Not breastfed	32.6	41.6	30.9	30.2	8.1(1)	29.7(1)	0.6(1)

Breastfed	67.4	58.4	69.1	69.8	0.004	0.0001	0.46

Total	100	100	100	100			

Missing values: maternal asthma none missing; year of birth none; mother's age 7 (0.2% of cases; social class 869 (27.6%*); marital status 8 (0.2%); parity 2 (0.05%); maternal smoking 1278 (31.8%*); gestational age 558 (13.9%); birth weight 114 (2.8%); caesarean section 114 (2.8%); forceps 114 (2.8%); Apgar 1 1164 (29.0%*); number of babies none missing; sex none; breastfeeding 961 (23.9%*). * = data item not collected in the first five years covered by the study. Numbers on which the percentages are based are given in additional files (see Additional file 1).

In addition, to address the issue that there may be greater diagnostic uncertainty with a diagnosis of asthma in people aged under 6 years than at 6 and over, we analysed the risk factors in those aged 6 and over at hospital admission. Risk factors in people admitted at 6 and over (see Additional file 2) were similar to those in people admitted at age 2 and over. Factors significantly associated with asthma in people aged 6 and over, in univariate analysis, were maternal asthma, maternal age (asthma less common in children of mothers aged 25-34 than mothers who were younger or older), low social class, maternal smoking in pregnancy, short gestational age, caesarean delivery and male sex.

Many of the perinatal factors were themselves inter-related (e.g. maternal age, social class, maternal smoking, birth weight). In the multivariate model (Table 3), the factors that were associated with a high risk of asthma, after taking account of all other significant factors in the model (which included year of birth), were maternal asthma (OR 3.1; 95% confidence interval 2.7-3.6; Table 3), male sex (1.8; 1.7-2.0), and low birth weight (1000-2999 g versus 3000-3999 g, 1.2; 1.1-1.3); and high social class was protective (1 and 2, compared with 3, 0.8; 0.7-0.9). Each of these effects was stronger in those first admitted when under 2 years of age than in those who were older. Factors associated with asthma but only in those under 2 years were maternal smoking during pregnancy (1.35 1.04-1.74), short gestational age after allowing for low birth weight (24-37 weeks versus 38-41 wks, 1.6; 1.2-2.2), and previous children (e.g. 1 versus 0, 1.3; 1.0-1.7). Caesarean section was significantly associated with asthma overall, in multivariate analysis, but was not significant in either age group individually. The division into age groups does, of course, reduce the numbers and therefore the statistical power in each individual age group.

**Table 3:** Results of multivariate model of best fit, derived through logistic regression, showing odds ratios (OR) with 95% confidence intervals, for the factors with significant and independent effects on the likelihood of developing asthma.

		All asthma		Aged 0-1 at asthma admission		Aged 2+ at asthma admission	
**Characteristics**		**OR**	**95% C.I**.	**OR**	**95% C.I**.	**OR**	**95% C.I**

							

Maternal asthma	No	1.00	-	1.00	-	1.00	-

	Yes	3.11	2.67-3.63	3.89	2.71-5.57	3.07	2.61-3.60

Social class	1-2	0.81	0.74-0.89	0.76	0.58-0.98	0.81	0.74-0.89

	3	1.00	-	1.00	-	1.00	-

	4-5	1.07	0.96-1.20	1.09	0.82-1.47	1.04	0.94-1.16

Sex	Female	1.00	-	1.00	-	1.00	-

	Male	1.82	1.66-1.99	2.08	1.64-2.65	1.53	1.41-1.66

Birth weight (g)	1000-2999	1.20	1.09-1.33	1.42	1.08-1.86	1.21	1.10-1.33

	3000-3999	1.00		1.00		1.00	-

	4000-5499	1.01	0.87-1.17	0.88	0.58-1.34	1.06	0.92-1.22

Gestation age (wks)	24-37	-	-	1.59	1.15-2.20	-	-

	38-41	-	-	1.00	-	-	-

	42-47	-	-	1.28	0.89-1.83	-	-

Smoking	No	1.00	-	1.00	-	-	-

	Yes	1.13	1.02-1.25	1.35	1.04-1.74	-	-

Parity	0	-	-	1.00	-	-	-

	1			1.34	1.03-1.74		

	2			1.38	0.99-1.93		

	3			2.19	1.42-3.38		

	4+	-	-	2.01	1.07-3.76	-	-

Caesarean	No	1.00	-	-	-	-	-

	Yes	1.18	1.02-1.34	-	-	-	-

All variables that were significant (p < 0.05) in the univariate analysis (see Table 2) were included in the initial model and the variables that were not significant were removed. In further modelling, each variable that was not significant in univariate analysis was re-introduced, one at a time, into the model. The purpose of this was to test whether any variable, not significant in univariate analysis, became so when modelled with the other significant variables.

In the multivariate model for children admitted to hospital with asthma when aged 6 or older, the factors associated with a high risk of asthma were similar to those for the overall model: significant associations were found with maternal asthma (OR 3.8; 3.1-4.7), age of mother (under 25 at birth, compared with 25-34 at birth, OR 1.16; 1.03-1.31; over 35 at birth, compared with 25-34 at birth, OR 1.4; 1.1-1.7); and high social class was protective (1 and 2, compared with 3, 0.7; 0.6-0.8). Hospital admissions for asthma in people aged over six was more common in males than females (1.4; 1.2-1.5); but, by the teenage years, the sex ratio reversed (Table 1) and admission was more common in females than males.

## Discussion

A major strength of this analysis is that it was undertaken in a large, defined population that covered over 30 years of data collection and almost a quarter of a million births. Another important strength is that information about perinatal risk factors and about the main outcome measure - asthma in the child - were originally recorded and collected independently of one another. They were subsequently brought together by record linkage. This means that data about each risk factor could not have been influenced by the presence or absence of the outcome measure. The study is therefore not subject to potential biases, such as interviewer and recall bias, that can affect interview-based case control studies. We also hope that the addition of this large study to the literature will be helpful to future researchers, interested in summarising studies on perinatal factors and asthma, who may seek data for meta-analysis.

Our age groups are those at first admission; and it is likely that some of the first admissions at older ages were for children who already had asthma, without hospitalisation, at a younger age. We were not able to identify data about resident children who may have been admitted to hospital outside the ORLS area or about children who were admitted with asthma after moving home to outside the ORLS area. Another limitation of our data is that it is confined to hospital admission and day case care for asthma. In general, this means that our findings are likely to be relevant to those whose asthma is severe enough to warrant hospital admission at least once. The possibility of bias through selective referral to hospital must also be considered. It is possible, for example, that some of the effects shown by us are associations with thresholds for admission for asthma as well as (or instead of) associations with asthma itself. However, our findings are generally similar to those reported recently in a large record linkage study from Finland that was not confined to hospitalisation data [2]. In the Finnish study, children and mothers with asthma were identified from prescription data for anti-asthmatic drugs rather than from hospital records. They reported on children with asthma under the age of 3 years. Comparing their findings with ours on children under 2, the Finnish study showed an odds ratio for maternal asthma of 3.41 (ours was 3.89), that 64% of the children with asthma were male (our figure was 63%), an odds ratio with maternal smoking in pregnancy of 1.35 (ours was 1.33), and both they and we found an association between elevated rates of asthma and birth by caesarian section, and a protective association with being first-born, normal gestational age and normal vaginal delivery.

The finding on maternal asthma adds to the evidence that there is a familial predisposition to asthma in children of those with asthma [2,3,10]. It is possible that maternal asthma may lead to reduced oxygenation of the foetus during development, which in turn may affect asthma development in the child [11]. It is also likely, of course, that some of the familial association reflects genetic factors affecting asthma risk. It is conceivable that mothers with asthma may be more likely than others to seek hospital admission for children with symptoms of asthma though, whilst this might have affected our study, it could not have influenced the Finnish result [2]. Shared environmental factors between mother and child would also play a role in the family association.

Our finding on the risk of maternal smoking for asthma in young children is consistent with similar studies performed in Finland and Sweden [2,12]. Most studies [4,10,13] but not all [14] have reported maternal smoking as a risk factor for childhood asthma. Our data only cover smoking in pregnancy. We cannot distinguish between any effect on the foetus and any effect on the child of mothers whose smoking continued after birth.

The increase in asthma across the birth cohorts of the 1970s and 1980s adds further weight to the now well established evidence that the incidence of asthma increased in children from the 1970s through the 1980s [15].

An association with low birth weight has been reported in most previous studies on perinatal risk factors for asthma [2,4,5,10,16]. Most previous studies have found low birth weight and/or short gestational age to be risk factors for asthma [2,4,5,7,10,12,16].

The reporting of an effect of older siblings on the risk of asthma varies from study to study. We found an increased risk of asthma in young children with older siblings (using parity of the mother as a proxy for having older siblings), as have others [2,12,17].

Our study adds to the evidence that there may be a link between caesarean delivery and an increased risk of asthma in young children [8]. Although we cannot distinguish between planned and emergency caesarean section, both were associated with a higher risk of asthma in children under the age of three in the Finnish study [2]. Caesarean delivery has been shown to alter and delay the development of certain intestinal bacterial flora, compared to vaginal delivery. This may affect the risk of atopic disease in children by altering immune development [8,18]. Delayed removal of amniotic fluid from the lungs of babies born by caesarean delivery, as compared with removal in the course of vaginal delivery, may also be important [18]. The possibility that caesarian section may increase the risk of asthma in young children, if confirmed, is a reason for discouraging caesarian section unless clinically necessary.

Admission for asthma was less common in children in high social class families than in others. We cannot distinguish between a direct association between class and asthma or whether children in higher social classes are less likely than others to be admitted to hospital with similar severity of symptoms. However, the difference found was between social classes 1 and 2, on the one hand, and 3, 4 and 5, on the other; and social class 3 is a large group of the population (45% of all children in Table 2). Any decision to admit a child for social reasons, when a child with similar symptoms would normally be treated at home, is likely to be associated with a greater extreme of low socio-economic status than that of a typical social class 3 (or 4) family. We incline to the interpretation that severe asthma may indeed have been a little less common in social classes 1 and 2.

The negative association we found between breastfeeding and subsequent asthma in very young children did not remain significant after multivariate analysis. Although a meta-analysis has reported a decreased risk of asthma in young children who were exclusively breastfed for the first 3 months of life [9], the evidence does not seem conclusive [19,20]. The effect of breastfeeding remains uncertain and is likely to depend on duration of breastfeeding which we could not identify.

## Conclusions

Our study adds to the evidence that the following factors have significant and independent effects on the risk of asthma: maternal asthma, male sex in childhood, low birth weight and short gestational age, maternal smoking, delivery by caesarean section, and having older siblings. The study also demonstrates the utility of using routinely collected health data, with data linkage, for studying potential perinatal risk factors for diseases in the child.

## Competing interests

The authors declare that they have no competing interests.

## Authors' contributions

RD and MJG designed the study. CJW undertook the analyses. SER  advised on the analyses. All authors contributed to the interpretation and discussion of findings. RD wrote the first draft and all authors contributed to the final manuscript.

## Pre-publication history

The pre-publication history for this paper can be accessed here:

http://www.biomedcentral.com/1471-2466/10/14/prepub

## Supplementary Material

Additional file 1 **Number of asthma cases by age at admission (0-1, and 2+)**. Numbers on which the percentages in Table 2 are based Click here for file

Additional file 2 **Percentage distribution of values of each factor in children aged 6 or older at hospital admission for asthma, with chi-square tests of association**. Percentage distribution of values of each factor in children aged 6 or older at hospital admission for asthma. Click here for file

## References

[B1] Eder W Ege MJ von Mutius E The asthma epidemic N Engl J Med 2006 355 2226 2235 10.1056/NEJMra054308 17124020

[B2] Metsälä J Kilkkinen A Kaila M Tapanainen H Klaukka T Gissier M Virtanen SM Perinatal factors and the risk of asthma in childhood - A population-based register study in Finland Am J Epidemiol 2008 168 170 178 10.1093/aje/kwn105 18511427

[B3] Berz JB Carter AS Wagmiller RL Horwitz SM Murdock KK Briggs-Gowan M Prevalence and correlates of early onset asthma and wheezing in a healthy birth cohort of 2- to 3- year olds J Pediatr Psychol 2007 32 154 166 10.1093/jpepsy/jsj123 16690752

[B4] Jaakkola JJK Gissler M Maternal smoking in pregnancy, fetal development and childhood asthma Am J Public Health 2004 94 136 140 10.2105/AJPH.94.1.136 14713711 PMC1449839

[B5] Dik N Tate RB Manfreda J Anthonisen NR Risk of physician-diagnosed asthma in the first 6 years of life Chest 2004 126 1147 1153 10.1378/chest.126.4.1147 15486376

[B6] Schaubel D Johansen H Dutta M Desmeules M Becker A Mao Y Neonatal characteristics as risk factors for preschool asthma J Asthma 1996 33 255 264 10.3109/02770909609055366 8707780

[B7] Jaakkola JJK Ahmed P Ieromnimon A Goepfert P Laiou E Quansah R Jaakkola MS Preterm delivery and asthma: a systematic review and meta-analysis J Allergy Clin Immunol 2006 118 823 830 10.1016/j.jaci.2006.06.043 17030233

[B8] Thavagnanam S Fleming J Bromley A Shields MD Cardwell CR A meta-analysis of the association between Caesarean section and childhood asthma Clin Exp Allergy 2008 38 629 633 10.1111/j.1365-2222.2007.02780.x 18352976

[B9] Gdalevich M Mimouni D Mimouni M Breast-feeding and the risk of bronchial asthma in childhood: a systematic review with meta-analysis of prospective studies J Pediatr 2001 139 261 266 10.1067/mpd.2001.117006 11487754

[B10] Oliveti JF Kercsmar CM Redline S Pre- and perinatal risk factors for asthma in inner city African-American children Am J Epidemiol 1996 143 570 577 8610674 10.1093/oxfordjournals.aje.a008787

[B11] Bracken MB Belanger K Cookson WO Triche E Christiani DC Leaderer BP Genetic and perinatal risk factors for asthma onset and severity: a review and theoretical analysis Epidemiol Rev 2002 24 176 189 10.1093/epirev/mxf012 12762091

[B12] Bråbäck L Björ O Nordahl G Early determinants of first hospital admissions for asthma and acute bronchitis among Swedish children Acta Paediatr 2003 92 27 33 10.1080/08035320310000410 12650295

[B13] Räsänen M Kaprio J Laitinen T Winter T Koskenvuo M Laitinen LA Perinatal risk factors for asthma in Finnish adolescent twins Thorax 2000 55 25 31 10.1136/thorax.55.1.25 10607798 PMC1745589

[B14] Gilman SE Gardener H Buka SL Maternal smoking during pregnancy and children's cognitive and physical development: a causal risk factor? Am J Epidemiol 2008 168 522 531 10.1093/aje/kwn175 18653646 PMC2597003

[B15] Anderson HR Gupta R Strachan DP Limb ES 50 years of asthma: UK trends from 1955 to 2004 Thorax 2007 62 85 90 10.1136/thx.2006.066407 17189533 PMC2111282

[B16] Kiechl-Kohlendorfer U Horak E Mueller W Strobl R Haberland C Fink FM Schwaiger M Gutenberger KH Reich H Meraner D Kiechl S Neonatal characteristics and risk of atopic asthma in schoolchildren: results from a large prospective birth-cohort study Acta Paediatr 2007 96 1606 1610 10.1111/j.1651-2227.2007.00449.x 17888060

[B17] McKeever TM Lewis SA Smith C Collins J Heatlie H Frischer M Hubbard R Siblings, multiple births, and the incidence of allergic disease: a birth cohort study using the West Midlands general practice research database Thorax 2001 56 758 762 10.1136/thorax.56.10.758 11562513 PMC1745942

[B18] Salam MT Margolis HG McConnell R McGregor JA Avol EL Gilliland FD Mode of delivery is associated with asthma and allergy occurrences in children Ann Epidemiol 2006 16 341 346 10.1016/j.annepidem.2005.06.054 16242961

[B19] Sears MR Greene JM Willan AR Taylor R Flannery EM Cowan JO Herbison P Poulton R Long-term relation between breastfeeding and development of atopy and asthma in children and young adults: a longitudinal study Lancet 2002 360 901 907 10.1016/S0140-6736(02)11025-7 12354471

[B20] Takemura Y Sakurai Y Honjo S Kusakari A Hara T Gibo M Tokimatsu A Kugai N Relation between breastfeeding and the prevalence of asthma: The Tokorozawa childhood asthma and pollinosis study Am J Epidemiol 2001 154 115 119 10.1093/aje/154.2.115 11447043

