# Supplementary material for: Influence of maternal and perinatal factors on subsequent hospitalisation for asthma in children: evidence from the Oxford record linkage study
Source: BMC Pulm Med. 2010 Mar 16;10:14. doi: 10.1186/1471-2466-10-14 (PMC2846893; doi:10.1186/1471-2466-10-14)
Supplement: Additional file 1 — Number of asthma cases by age at admission (0-1, and 2+). Numbers on which the percentages in Table 2 are based [file 1471-2466-10-14-S1.DOC]

**Additional file 2: Number of asthma cases by age at admission (0-1, and 2+).**

|  |  | **Number of asthma cases** | |  |
| --- | --- | --- | --- | --- |
|  | **0-1** | **2+** | **No asthma** | **Total** |
| **Maternal asthma:** |  |  |  |  |
| **No** | 487 | 3209 | 238620 | 242316 |
| **Yes** | 48 | 272 | 5928 | 6248 |
| **Total** | 535 | 3481 | 244548 | 248564 |
|  |  |  |  |  |
| **Year of birth:** |  |  |  |  |
| **1970-1974** | 22 | 736 | 68181 | 68939 |
| **1975-1979** | 41 | 829 | 55611 | 56481 |
| **1980-1984** | 124 | 1018 | 59220 | 60362 |
| **1985-1989** | 348 | 899 | 61583 | 62830 |
| **Total** | 535 | 3482 | 244595 | 248612 |
|  |  |  |  |  |
| **Maternal age:** |  |  |  |  |
| **14-24** | 206 | 1289 | 85038 | 86533 |
| **25-34** | 296 | 1908 | 140702 | 142906 |
| **35-49** | 33 | 278 | 18538 | 18849 |
| **Total** | 5354 | 3475 | 244278 | 248288 |
|  |  |  |  |  |
| **Social class:** |  |  |  |  |
| **I + II** | 115 | 835 | 67280 | 68230 |
| **III** | 186 | 1331 | 85332 | 86849 |
| **IV + V** | 86 | 595 | 34823 | 35504 |
| **Total** | 387 | 2761 | 187435 | 190583 |
|  |  |  |  |  |
| **Marital status:** |  |  |  |  |
| **Married** | 424 | 3105 | 220688 | 224217 |
| **Not married** | 110 | 370 | 23456 | 23936 |
| **Total** | 534 | 3475 | 244144 | 248153 |
|  |  |  |  |  |

|  |  | **Number of asthma cases** | |  |
| --- | --- | --- | --- | --- |
|  | **0-1** | **2+** | **No asthma** | **Total** |
| **Parity of mother** |  |  |  |  |
| **0** | 190 | 1435 | 102566 | 104191 |
| **1+** | 345 | 2045 | 141796 | 144186 |
| **Total** | 536 | 3480 | 244362 | 248377 |
|  |  |  |  |  |
| **Maternal smoking** |  |  |  |  |
| **No** | 302 | 1661 | 108967 | 1109130 |
| **Yes** | 175 | 601 | 33463 | 34239 |
| **Total** | 477 | 2262 | 142430 | 145169 |
|  |  |  |  |  |
| **Gestational age (wks):** |  |  |  |  |
| **24-37** | 78 | 365 | 21462 | 21905 |
| **38-41** | 307 | 2369 | 171159 | 173835 |
| **42-47** | 49 | 291 | 20224 | 20564 |
| **Total** | 434 | 3025 | 212845 | 216304 |
|  |  |  |  |  |
| **Birth weight:** | 171 | 913 | 57451 | 58535 |
| **1000-2999** | 330 | 2251 | 1665545 | 168126 |
| **3000-3999** | 33 | 3099 | 20803 | 21145 |
| **4000-5499** | 534 | 3473 | 243799 | 247806 |
| **Total** |  |  |  |  |
|  |  |  |  |  |
| **Caesarian section:** |  |  |  |  |
| **No** | 447 | 3104 | 220202 | 223753 |
| **Yes** | 51 | 301 | 17667 | 18019 |
| **Total** | 498 | 3405 | 237869 | 241772 |
|  |  |  |  |  |

|  |  | **Number of asthma cases** | |  |
| --- | --- | --- | --- | --- |
|  | **0-1** | **2+** | **No asthma** | **Total** |
| **Forceps delivery:** |  |  |  |  |
| **No** | 465 | 2977 | 206617 | 210059 |
| **Yes** | 33 | 428 | 31252 | 31713 |
| **Total** | 498 | 3405 | 237869 | 241772 |
|  |  |  |  |  |
| **Apgar 1:** |  |  |  |  |
| **1-5** | 48 | 262 | 21042 | 21353 |
| **6-8** | 162 | 963 | 63332 | 64457 |
| **9-10** | 307 | 1966 | 137963 | 140236 |
| **Total** | 518 | 3190 | 222337 | 226045 |
|  |  |  |  |  |
| **Apgar 5:** |  |  |  |  |
| **1-5** | 5 | 14 | 864 | 883 |
| **6-8** | 18 | 74 | 4136 | 4228 |
| **9-10** | 455 | 2287 | 146048 | 148790 |
| **Total** | 478 | 2375 | 151048 | 153901 |
|  |  |  |  |  |
| **Number of babies delivered:** |  |  |  |  |
| **1** | 522 | 3420 | 239282 | 243224 |
| **2+** | 13 | 62 | 5313 | 5388 |
| **Total** | 535 | 3482 | 244595 | 248612 |
|  |  |  |  |  |
| **Head circumference:** |  |  |  |  |
| **<34** | 125 | 530 | 34020 | 34675 |
| **34-34.9** | 117 | 568 | 38431 | 39116 |
| **35-35.9** | 109 | 859 | 37851 | 38819 |
| **36+** | 149 | 530 | 50004 | 50683 |
| **Total** | 500 | 2519 | 160306 | 163325 |
|  |  |  |  |  |

|  |  | **Number of asthma cases** | |  |
| --- | --- | --- | --- | --- |
|  | **0-1** | **2+** | **No asthma** | **Total** |
| **Sex of baby:** |  |  |  |  |
| **Female** | 161 | 1340 | 119296 | 120797 |
| **Male** | 374 | 2142 | 125292 | 127808 |
| **Total** | 535 | 3482 | 244588 | 248605 |
|  |  |  |  |  |
| **Breastfed or not:** |  |  |  |  |
| **Not breastfed** | 204 | 793 | 49957 | 50954 |
| **Breastfed** | 287 | 1772 | 115270 | 117329 |
| **Total** | 491 | 2565 | 165227 | 168283 |
|  |  |  |  |  |
