# Supplementary material for: Influence of maternal and perinatal factors on subsequent hospitalisation for asthma in children: evidence from the Oxford record linkage study
Source: BMC Pulm Med. 2010 Mar 16;10:14. doi: 10.1186/1471-2466-10-14 (PMC2846893; doi:10.1186/1471-2466-10-14)
Supplement: Additional file 2 — Percentage distribution of values of each factor in children aged 6 or older at hospital admission for asthma, with chi-square tests of association. Percentage distribution of values of each factor in children aged 6 or older at hospital admission for asthma. [file 1471-2466-10-14-S2.DOC]

**Additional file 1: Percentage distribution of values of each factor in children aged 6+ with asthma and in children aged 6+ with no asthma, with chi-square tests of association.**

|  |  | **Asthma aged 6+ (N=1699)** | | |  | **No asthma aged 6+ (N=246960)** | | |  |  |
| --- | --- | --- | --- | --- | --- | --- | --- | --- | --- | --- |
|  |  |  |  |  |
|  |  |  |  |  |  |  |  |  |  | **Chi sq** |
|  |  | **n** |  | **%** |  | **N** |  | **%** |  | **p value** |
|  |  |  |  |  |  |  |  |  |  |  |
| **Maternal asthma:** |  |  |  |  |  |  |  |  |  |  |
| **No** |  | 1563 |  | 92.0 |  | 240799 |  | 97.5 |  | 210.6 (1) |
| **Yes** |  | 136 |  | 8.0 |  | 6112 |  | 2.5 |  | <0.0001 |
| **Total** |  | 1699 |  | 100 |  | 246911 |  | 100 |  |  |
|  |  |  |  |  |  |  |  |  |  |  |
| **Maternal age:** |  |  |  |  |  |  |  |  |  |  |
| **14-24** |  | 667 |  | 39.4 |  | 85877 |  | 34.8 |  | 19.4 (2) |
| **25-34** |  | 886 |  | 52.3 |  | 142053 |  | 57.6 |  | <0.0001 |
| **35-49** |  | 141 |  | 8.3 |  | 18711 |  | 7.6 |  |  |
| **Total** |  | 1694 |  | 100 |  | 246641 |  | 100 |  |  |
|  |  |  |  |  |  |  |  |  |  |  |
| **Social class:** |  |  |  |  |  |  |  |  |  |  |
| **I + II** |  | 366 |  | 26.9 |  | 67878 |  | 35.9 |  | 56.2 (2) |
| **III** |  | 669 |  | 49.1 |  | 86200 |  | 45.6 |  | <0.0001 |
| **IV + V** |  | 328 |  | 24 |  | 35182 |  | 18.6 |  |  |
| **Total** |  | 1363 |  | 100 |  | 189260 |  | 100 |  |  |
|  |  |  |  |  |  |  |  |  |  |  |
| **Marital status:** |  |  |  |  |  |  |  |  |  |  |
| **Married** |  | 1542 |  | 90.9 |  | 222719 |  | 90.4 |  | 0.6 (1) |
| **Not married** |  | 154 |  | 9.1 |  | 23785 |  | 9.6 |  | 0.43 |
| **Total** |  | 1696 |  | 100 |  | 246504 |  | 100 |  |  |
|  |  |  |  |  |  |  |  |  |  |  |
| **Parity of mother:** |  |  |  |  |  |  |  |  |  |  |
| **0** |  | 692 |  | 40.7 |  | 103518 |  | 42.0 |  | 4.9 (4) |
| **1** |  | 620 |  | 36.5 |  | 88461 |  | 35.9 |  | 0.3 |
| **2** |  | 245 |  | 14.4 |  | 35873 |  | 14.5 |  |  |
| **3** |  | 81 |  | 4.8 |  | 12029 |  | 4.9 |  |  |
| **4+** |  | 61 |  | 3.6 |  | 6844 |  | 2.8 |  |  |
| **Total** |  | 1699 |  | 100 |  | 246725 |  | 100 |  |  |
|  |  |  |  |  |  |  |  |  |  |  |
| **Maternal smoking:** |  |  |  |  |  |  |  |  |  |  |
| **No** |  | 653 |  | 72.9 |  | 110308 |  | 76.4 |  | 6.3 (1) |
| **Yes** |  | 243 |  | 27.1 |  | 34002 |  | 23.6 |  | 0.01 |
| **Total** |  | 896 |  | 100 |  | 144310 |  | 100 |  |  |
|  |  |  |  |  |  |  |  |  |  |  |
| **Gestational age (wks):** |  |  |  |  |  |  |  |  |  |  |
| **24-37** |  | 181 |  | 12.2 |  | 21731 |  | 10.1 |  | 7.0 (2) |
| **38-41** |  | 1164 |  | 78.3 |  | 172704 |  | 80.4 |  | 0.03 |
| **42-47** |  | 141 |  | 9.5 |  | 20426 |  | 9.5 |  |  |
| **Total** |  | 1486 |  | 100 |  | 214861 |  | 100 |  |  |
|  |  |  |  |  |  |  |  |  |  |  |
| **Birth weight:** |  |  |  |  |  |  |  |  |  |  |
| **1000-2999** |  | 426 |  | 25.1 |  | 58127 |  | 23.6 |  | 2.7 (2) |
| **3000-3999** |  | 1119 |  | 66.0 |  | 167030 |  | 67.8 |  | 0.26 |
| **4000-5499** |  | 150 |  | 8.9 |  | 21001 |  | 8.5 |  |  |
| **Total** |  | 1695 |  | 100 |  | 246158 |  | 100 |  |  |
|  |  |  |  |  |  |  |  |  |  |  |
| **Caesarean section:** |  |  |  |  |  |  |  |  |  |  |
| **No** |  | 1533 |  | 91.2 |  | 222260 |  | 92.6 |  | 4.5 (1) |
| **Yes** |  | 148 |  | 8.8 |  | 17877 |  | 7.4 |  | 0.03 |
| **Total** |  | 1681 |  | 100 |  | 240137 |  | 100 |  |  |
|  |  |  |  |  |  |  |  |  |  |  |
| **Forceps delivery:** |  |  |  |  |  |  |  |  |  |  |
| **No** |  | 1438 |  | 85.5 |  | 208662 |  | 86.9 |  | 2.7 (1) |
| **Yes** |  | 243 |  | 14.5 |  | 31475 |  | 13.1 |  | 0.1 |
| **Total** |  | 1681 |  | 100 |  | 240137 |  | 99.3 |  |  |
|  |  |  |  |  |  |  |  |  |  |  |
| **Apgar 1:** |  |  |  |  |  |  |  |  |  |  |
| **1-5** |  | 136 |  | 8.9 |  | 21220 |  | 9.5 |  | 4.5 (2) |
| **6-8** |  | 475 |  | 30.9 |  | 63994 |  | 28.5 |  | 0.1 |
| **9-10** |  | 925 |  | 60.2 |  | 139342 |  | 62.0 |  |  |
| **Total** |  | 1536 |  | 100 |  | 224556 |  | 100 |  |  |
|  |  |  |  |  |  |  |  |  |  |  |
| **Number of babies in birth delivery:** |  |  |  |  |  |  |  |  |  |  |
| **Singleton** |  | 1671 |  | 98.4 |  | 241598 |  | 97.8 |  | 2.2 (1) |
| **Multiple birth** |  | 28 |  | 1.6 |  | 5362 |  | 2.2 |  | 0.14 |
| **Total** |  | 1699 |  | 100 |  | 246960 |  | 100 |  |  |
|  |  |  |  |  |  |  |  |  |  |  |
| **Sex of baby:** |  |  |  |  |  |  |  |  |  |  |
| **Female** |  | 693 |  | 40.8 |  | 120130 |  | 48.6 |  | 41.7 (1) |
| **Male** |  | 1006 |  | 59.2 |  | 126823 |  | 51.4 |  | <0.0001 |
| **Total** |  | 1699 |  | 100 |  | 246953 |  | 100 |  |  |
|  |  |  |  |  |  |  |  |  |  |  |
| **Breastfed or not:** |  |  |  |  |  |  |  |  |  |  |
| **Not breastfed** |  | 354 |  | 32.6 |  | 50612 |  | 30.3 |  | 2.8 (1) |
| **Breastfed** |  | 732 |  | 67.4 |  | 116632 |  | 69.7 |  | 0.1 |
| **Total** |  | 1086 |  | 100 |  | 167244 |  | 100 |  |  |

Missing values in those aged 6 years or more: maternal asthma, none; year of birth, none; mother’s age, 5/1699 (0.3% of cases); social class, 336/1699 (19.8%*); marital status, 3/1699 (0.2%); parity, none; maternal smoking, 293/1189 (24.6%*); gestational age, 213/1699 (12.5%); birth weight, 4/1699 (0.24%); caesarean section, 18/1699 (1.1%); forceps, 18/1699 (1.1%); Apgar 1, 163/1699 (9.6%*); number of babies, none; sex, none; breastfeeding, 613/1699 (36.1%*). Missing values in full dataset listed as footnote on Table 2.

* Data item not collected in the first five years covered by the study.
